# Potential of the non-weight-bearing tunnel view in diagnosing medial meniscus posterior root tear: a pilot study of X-ray characteristics

**DOI:** 10.1186/s40634-021-00421-3

**Published:** 2021-10-30

**Authors:** Hiroki Okamura, Hiroki Ishikawa, Takuya Ohno, Shogo Fujita, Shigeo Yamakami, Hirotaka Akezuma, Koji Ishikawa, Katsunori Inagaki

**Affiliations:** 1 Department of Orthopaedic Surgery, Nihon Koukan Hospital, 1-2-1 Koukandori, Kawasaki-ku, Kawasaki City, Kanagawa Prefecture 210-0852 Japan; 2 Department of Orthopaedic Surgery, Showa University School of Medicine, Tokyo, 142-8555 Japan

**Keywords:** Medial meniscus posterior root tear, Non-weight-bearing tunnel view, Imaging, Open-wedge high-tibial osteotomy, Osteoarthritis

## Abstract

**Purpose:**

Early detection of medial meniscus posterior root tear (MMPRT) is important in preventing the rapid onset and progression of degenerative knee disease. Diagnosis is facilitated by the availability of non-weight-bearing X-ray view, but information on the X-ray characteristics of MMPRT is scarce. Here, we conducted a pilot study of the X-ray characteristics of MMPRT on non-weight-bearing tunnel view.

**Methods:**

We retrospectively reviewed 43 consecutive patients treated in the outpatient department for medial knee pain or popliteal pain. Patients were divided into MMPRT (21 knees) and non-MMPRT groups (22 knees). We investigated X-ray characteristics and magnetic resonance imaging findings. Femorotibial angle, posterior tibial slope, medial tibial eminence (MTE)–medial femoral condyle (MFC) distance (contralateral and affected sides, and difference between the two), medial tibiofemoral joint (MTFJ) width (contralateral and affected sides, and difference between the two), and meniscus radial dislocation between the groups were evaluated using the Mann–Whitney *U* test. The association between X-ray characteristics and MMPRT was determined using univariate and multivariate logistic regression analyses.

**Results:**

A highly significant difference between the affected and contralateral sides was seen in MTFJ width and MTE–MFC distance on non-weight-bearing tunnel view between the MMPRT and non-MMPRT groups. Moreover, a difference in MTFJ width of <−0.575 mm and in MTE–MFC distance of >0.665 mm between the affected and contralateral sides was useful in predicting MMPRT.

**Conclusions:**

The non-weight-bearing tunnel view is useful for the initial diagnosis of MMPRT. Prospective evaluation in a larger population is warranted.

## Background

Medial meniscus posterior root tear (MMPRT), defined as a radial tear <10 mm from the root attachment, widely perturbs the structure and function of the knee joint, and rapidly progresses to degenerative knee disease if left untreated. Early detection and treatment of MMPRT is therefore critical to avoiding this outcome [5, 6, 12, 23]. The medial meniscus serves important biomechanical functions, including shock absorption, joint stabilization, lubrication, and proprioception [21, 30]. The biomechanical consequences of MMPRT include disruption of the hoop-strain mechanism and an increase in peak contact pressure in the knee joint, approximated after total meniscectomy by 25% [5]. Additionally, MMPRT may give rise to spontaneous osteonecrosis of the knee and rapid exacerbation of osteoarthritis [12, 23]. Good clinical results have been reported for some MMPRT treatment methods, including pull-out repair, suture anchor repair, and open-wedge high-tibial osteotomy [5, 6, 20].

Diagnostic methods of MMPRT include clinical symptoms, and physical and radiological examination. Among symptoms, painful popping at onset is common, and is useful for diagnosis [13]. Although conclusive diagnosis requires magnetic resonance imaging (MRI) [9], X-ray radiography is simpler and therefore useful in initial diagnosis. X-ray findings reported to date include early joint-space narrowing and varus deformity on weight-bearing [13, 18]. Recently, Kodama et al. [15] reported the usefulness of the Rosenberg view in MMPRT, namely the weight-bearing tunnel view. However, weight-bearing X-ray radiography is generally a burden for these patients, whose knee pain can be so serious that normal walking is not possible [3, 13]. We therefore hypothesized that the non-weight-bearing tunnel view would be useful. To date, however, a non-weight-bearing X-ray method for diagnosing MMPRT has not been reported.

Here, to investigate the feasibility of the non-weight-bearing tunnel view for initial X-ray diagnosis of MMPRT, we conducted a pilot study to characterize X-ray findings in these patients, including femorotibial angle (FTA) by total length of the lower limbs on weight-bearing, posterior tibial slope (PTS) by lateral view, medial tibial eminence (MTE)–medial femoral condyle (MFC) distance by non-weight-bearing tunnel view, and medial tibiofemoral joint (MTFJ) width. We also compared results with those for medial radial displacement (MRD) by MRI.

## Methods

### Study population

The study was conducted under a retrospective cross-sectional design. The medical records of 547 knees treated for medial knee pain or popliteal pain in the outpatient clinic at XXXX Hospital in XXXX, XXXX, between April 2020 and January 2021 were reviewed. Eligibility criteria were: (1) unilateral knee pain; (2) Kellgren–Lawrence classification grade ≤ 2; (3) age > 30 years; (4) FTA ≤ 180°; (5) available X-ray radiography records for the front, lateral, and tunnel views of both knees; and (6) receipt of MRI evaluation. Based on these initial criteria, 93 knees were eligible for analysis. We then excluded patients with a previous ligament and/or meniscal injury, previous fracture around the knee, rheumatoid arthritis, and a meniscus tear other than MMPRT diagnosed on MRI. Finally, 43 patients were included, and divided into MMPRT (21 knees, 48.8%) and non-MMPRT control groups (22 knees, 51.2%).

### FTA and PTS measurements

**Fig. 1:**
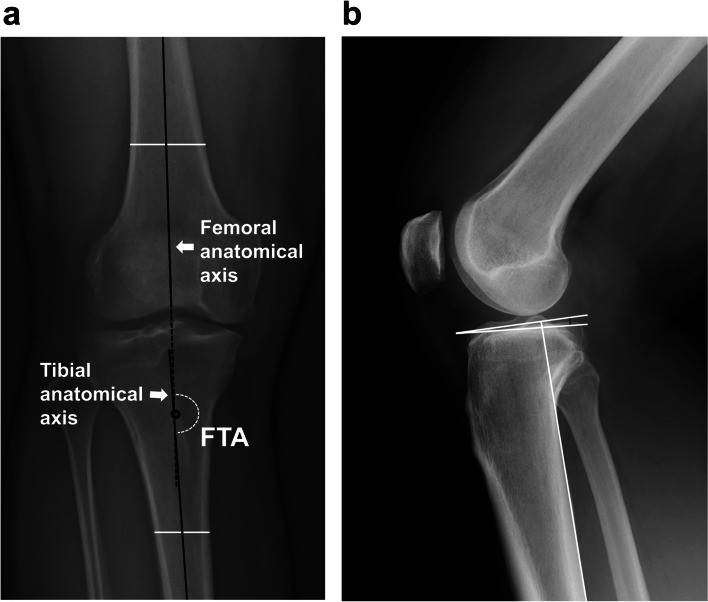
Femorotibial angle and posterior tibial slope measurements. **a** The femorotibial angle (FTA) is formed by two axes. The femoral anatomical axis originates from the femoral intercondylar notch point, and the tibial anatomical axis originates from the tibial plateau center. **b** The posterior tibial slope (PTS) was measured as the angle between the tangent to the medial tibial plateau and a line perpendicular to the posterior tibial cortex

The FTA was evaluated by the total length of the lower limbs on weight-bearing using digital tomography (Sonialvision G4; Shimadzu Medical Systems & Equipment, Japan) [31]. The knees were maximally extended and the patellae were facing forwards. The FTA is formed by two axes: the femoral and tibial anatomical axes. The femoral anatomical axis was defined as the line from the center of the femoral shaft 10 cm above the intercondylar notch to the intercondylar notch. The tibial anatomical axis was defined as the line from the center of the tibial shaft 10 cm below the tibial plateau to the tibial plateau center (Fig. 1) [11].

Special care was taken to ensure that the lateral view was taken in true lateral projection with overlapping femoral condyles. PTS was measured according to the Brazier method using the posterior tibial cortex perpendicular (Fig. 1) [7]. The posterior tibial cortex is easily identifiable on radiographs and serves as a reliable landmark, minimizing systematic error [10].

### Non-weight-bearing tunnel view measurement; MTE–MFC distance and MTFJ width

Several methods of obtaining the tunnel view have been reported, including the Holmland method (non-weight-bearing, 70° flexion), Béclère method (non-weight-bearing, 60° flexion), Camp–Coventry method (non-weight-bearing, 40°–50° flexion), Rosenberg method (weight-bearing, 45° flexion), and Schuss view (weight-bearing, 30°–40° flexion) [4]. In this study, the non-weight-bearing tunnel view was obtained with the knee flexed 60° over an angle block and sandbag, which provides an anteroposterior view with the patient supine. The X-ray beam was parallel to the tibial joint line.

**Fig. 2:**
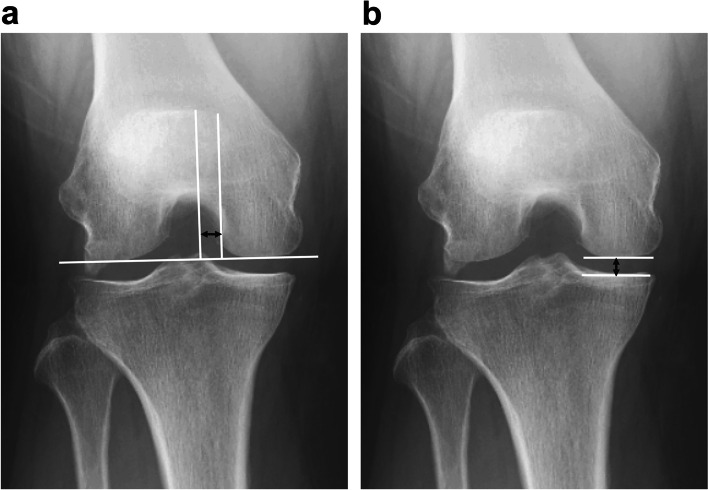
Non-weight-bearing tunnel view measurement; medial tibial eminence–medial femoral condyle distance; medial tibiofemoral joint width. **a** The medial tibial eminence (MTE) line was drawn perpendicular to the line tangent to the medial and lateral condyles. The medial femoral condyle (MFC) line is perpendicular to the medial and lateral condyles of the femur and tangent to the medial side of the femur. The MTE–MFC distance is indicated by a black arrow. **b** The medial tibiofemoral joint (MTFJ) width was measured from the center of the medial femoral condyle to the center of the medial tibial plateau. The MTFJ width is indicated by a black arrow

On this tunnel view, the MTE line was drawn perpendicular to the line tangent to the medial and lateral condyles. The MFC line was perpendicular to the medial and lateral condyles of the femur and tangent to the medial side of the femur. The MTE–MFC distance was measured using the Kodama method on the non-weight-bearing tunnel view (Fig. 2a) [15]. The MTFJ width was measured from the center of the MFC to the center of the medial tibial plateau (Fig. 2b) [15].

### MRD measurements on MRI

**Fig. 3:**
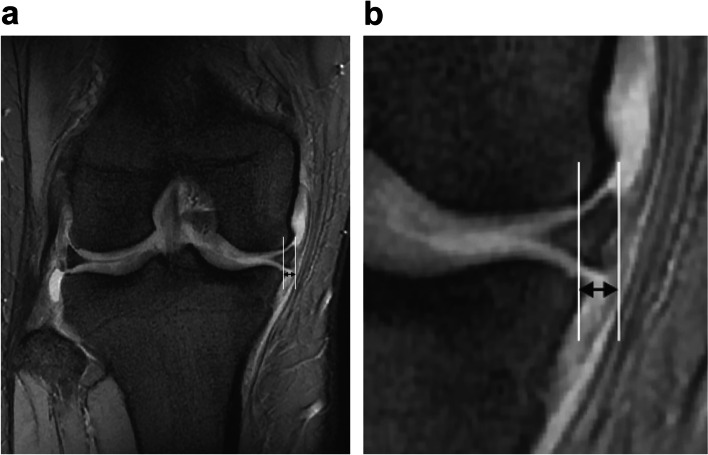
Medial radial displacement measurements on magnetic resonance imaging. **a** The medial radial displacement (MRD) measurement was first performed by drawing a vertical line on the medial edge of the tibial plateau. The length of another line extending from the first line to the medial edge of the meniscus was defined as the meniscal extrusion. The MRD is indicated by a black arrow. **b** Enlarged view of the part corresponding to the MRD in Fig 3**a**. The MRD is indicated by a black arrow

All knees were examined with a 1.5-T MRI scanner (SIGNA™ Voyager; GE Healthcare Japan Corp., Tokyo, Japan) using an 8-channel extremity coil in the axial, sagittal, and coronal planes. This study analyzed only coronal (T2*) scans at the level of the intercondylar eminence because the slice provides optimal delineation of the meniscus body [25, 28]. MRD measurement was first performed by drawing a vertical line on the medial edge of the tibial plateau. The length of a second line extending from the first line to the medial edge of the meniscus was defined as the meniscal extrusion (Fig. 3) [25]. Osteophytes were excluded when determining the medial edge of the tibial plateau [24].

### Statistical analysis

Continuous variables are presented as median and interquartile range (IQR). Differences in sex and affected side were evaluated using the chi-square test. As the measurement data was non-normally distributed, the differences in FTA (contralateral side, affected side), PTS (contralateral side, affected side), MTE–MFC distance (contralateral side, affected side, difference between contralateral and affected side), MTFJ width (contralateral side, affected side, difference between contralateral and affected side), and MRD between the MMPRT and non-MMPRT groups were evaluated using the Mann–Whitney *U* test. X-ray measurements were analyzed by both univariate and multivariate logistic analyses. Optimal cutoff values for the difference between the affected side and contralateral side in MTE–MFC distance and MTFJ width were calculated using a receiver operating characteristic (ROC) curve. The odds ratios (ORs) for MMPRT were calculated using multiple logistic regression analyses. All statistical analyses were performed using StatFlex version 7 (Artech Co., Ltd.). Covariates were selected for their ability to confound the association using univariate and stepwise models. All statistical tests were two-tailed, and *P*-values of <0.05 were considered to indicate statistical significance.

### Reproducibility

FTA, PTS, MTE–MFC distance, MTFJ width measurements, and MRD were assessed retrospectively by two orthopedists who were blinded to grouping. These assessments were repeated after a 2-week interval. To determine intra and interobserver reproducibility, these measurements were assessed using intra-class correlation coefficients (ICCs).

## Results

### Baseline patient characteristics

**Fig. 4:**
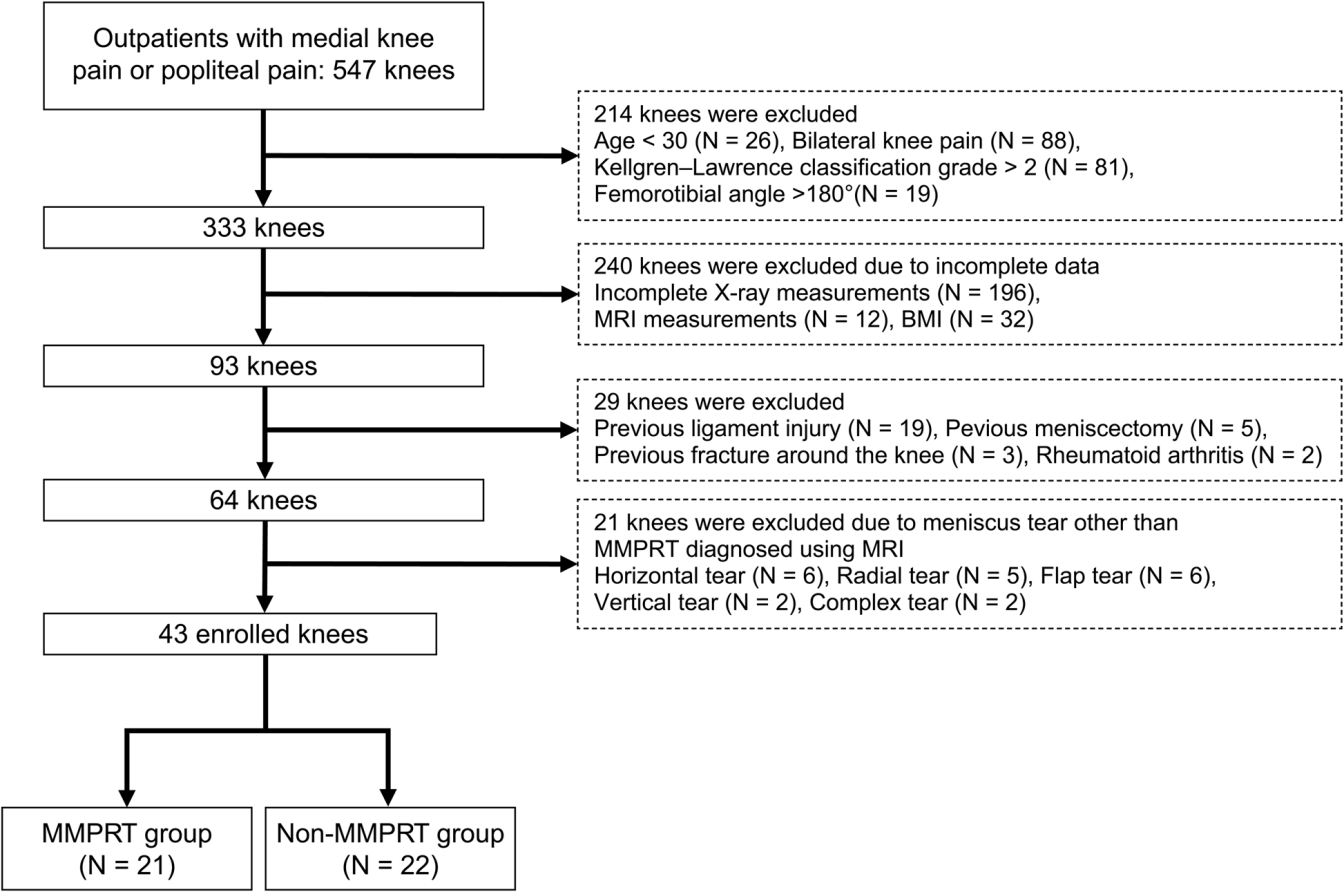
Flow diagram depicting the knee selection process in the study. MMPRT, medial meniscus posterior root tear; MRI, magnetic resonance imaging

A total of 43 knees were analyzed (MMPRT group, 21 knees, 48.8%; non-MMPRT group, 22 knees, 51.2%). Figure 4 shows a flow diagram of the patient selection process. Patient characteristics are presented in Table 1. Median age was 61.0 years (54.3–68.8 years), and median body mass index (BMI) was 25.5 kg/m^2^ (23.0–29.1 kg/m^2^). Median age in the MMPRT and non-MMPRT groups was 61.0 years (54.8–71.3 years) and 61.5 years (54.0–66.0 years) and median BMI was 26.3 (23.7–29.4) and 23.5 (22.7–27.0), respectively.

**Table 1:** Comparison of the patient characteristics between the MMPRT and non-MMPRT groups

	All (***n*** = 43)	MMPRT group (***n*** = 21)	Non-MMPRT group (***n*** = 22)	***P*** value
**Sex (male, female) (N)**	12, 31	4, 17	8, 14	0.21
**Affected side (right, left) (N)**	18, 25	7, 14	11, 11	0.26
**Age (years)**	61.0 (54.3–68.8)	61.0 (54.8–71.3)	61.5 (54.0–66.0)	0.67
**Weight (kg)**	62.0 (56.2–72.0)	61.0 (57.1–74.4)	66.5 (56.0–72.0)	0.97
**Height (cm)**	158.0 (152.7–166.8)	153.0 (148.8–161.7)	160.0 (157.0–167.1)	<0.01*
**BMI (kg/m**^**2**^**)**	25.5 (23.0–29.1)	26.3 (23.7–29.4)	23.5 (22.7–27.0)	0.06

*Note*: Data on age, weight, height, and BMI are expressed as median (IQR: Q1–Q3). *P < 0.05

*Abbreviations*: *MMPRT* medial meniscus posterior root tear, *IQR* interquartile range, *BMI* body mass index

### Comparison of X-ray and MRI measurements among patients in the MMPRT and non-MMPRT groups

Table 2 compares X-ray and MRI measurements between the MMPRT and non-MMPRT groups. Significant differences between the groups were seen in PTS (contralateral side and affected side), MTE–MFC distance (affected side and difference between affected side and contralateral side), MTFJ width (affected side and difference between affected side and contralateral side), and MRD.

**Table 2:** Comparison of X-ray and MRI measurements between patients in the MMPRT group and non-MMPRT group

	All (***n*** = 43)	MMPRT group (***n*** = 21)	Non-MMPRT group (***n*** = 22)	***P value***
**FTA**				
Contralateral side (°)	177.3 (176.0–178.8)	177.0 (176.0–178.4)	177.4 (176.3–179.3)	*0.39*
Affected side (°)	178.3 (176.8–179.0)	178.3 (177.1–179.3)	178.4 (175.8–179.0)	*0.42*
**PTS**				
Contralateral side (mm)	6.7 (5.3–8.2)	8.5 (7.3–10.8)	5.7 (4.5–7.3)	<0.01*
Affected side (mm)	7.3 (5.1–9.1)	8.5 (7.3–10.8)	5.7 (4.5–7.3)	<0.01*
**MTE–MFC distance**				
Contralateral side (mm)	5.1 (4.2–5.5)	5.1 (4.1–7.8)	5.0 (4.5–5.4)	*0.64*
Affected side (mm)	6.0 (5.0–7.3)	7.2 (6.2–7.9)	5.4 (4.4–6.0)	<0.01*
Difference between affected side and contralateral side (mm)	0.7 (0.2–2.1)	1.9 (0.8–2.6)	0.3 (−0.7–0.3)	<0.01*
**MTFJ width**				
Contralateral side (mm)	4.9 (4.3–5.3)	5.0 (4.3–5.4)	4.7 (4.3–5.2)	*0.36*
Affected side (mm)	4.1 (3.5–4.7)	3.8 (3.0–4.1)	4.5 (4.0–5.3)	<0.01*
Difference between affected side and contralateral side (mm)	−0.6 (−1.4–−0.1)	−1.2 (−2.3–−0.7)	−0.1 (−0.5–0.3)	<0.01*
**MRD (mm)**	3.5 (2.5–4.5)	4.1 (3.7–5.2)	2.5 (0.8–3.3)	<0.01*

*Note*: Data are expressed as median (IQR: Q1–Q3); Mann–Whitney *U* test. *P < 0.05

*Abbreviations*: *MRI* magnetic resonance imaging, *MMPRT* medial meniscus posterior root tear, *FTA* femorotibial angle, *PTS* posterior tibial slope, *MTE* medial tibial eminence, *MFC* medial femoral condyle, *MTFJ* medial tibial femoral joint, *MRD* medial radial displacement, *IQR* interquartile range

### Univariate and ROC curve analyses

Univariate logistic regression analysis revealed significant associations between MMPRT incidence and difference between the affected side and contralateral side in MTE–MFC distance and in MTFJ width (Table 3). To estimate the power of these X-ray measurements to predict MMPRT, areas under the ROC curves (AUCs) obtained using univariate logistic regression analysis were calculated. On ROC curve analysis, a difference between the affected and contralateral sides in MTE–MFC distance >0.665 mm was the strongest predictor (AUC = 0.829) of MMPRT. Using a cutoff value of 0.665 mm, the sensitivity and specificity for predicting MMPRT were 81.0% and 81.8%, respectively. Similarly, a difference between the affected and contralateral sides in MTFJ width <−0.575 mm (AUC = 0.872) was also a useful predictor of MMPRT.

**Table 3:** Univariate logistic regression analysis of X-ray measurements for MMPRT

X-ray measurements	OR (95% CI)	***P*** value
**Difference between the affected side and contralateral side in MTE–MFC distance (per 0.1 mm increase)**	1.140 (1.054–1.223)	0.001*
**Difference between the affected side and contralateral side in MTFJ width (per 0.1 mm decrease)**	1.121 (1.031–1.219)	0.008*

*Abbreviations*: *OR* Odds ratio, *CI* confidence interval, *MTE* medial tibial eminence, *MFC* medial femoral condyle, *MTFJ* medial tibial femoral joint

*Note*: *P < 0.05

### Multivariate logistic regression analyses for MMPRT

The results of the multivariate logistic regression analysis of X-ray measurements are shown in Table 4. On multivariate logistic regression analyses, a difference between the affected and contralateral side in MTE–MFC distance >0.665 and MTFJ width <−0.575 were independent predictors of MMPRT. Moreover, these significant ORs persisted after adjustment for age, BMI, and PTS of the affected side (difference between the affected and contralateral side in MTE–MFC distance >0.665 mm: OR = 15.38, 95% confidence interval (CI): 2.687–88.067, *P* = 0.002; and difference between the affected and contralateral side in MTFJ width <−0.575 mm: OR = 50.85, 95% CI: 3.648–708.604, *P* = 0.003).

**Table 4:** Multivariate logistic regression analysis of X-ray measurements for MMPRT

	OR (95% CI)	***P*** value	OR^**a**^ (95% CI)	***P*** value
**Difference between the affected side and contralateral side of the MTE–MFC distance (≤0.665 mm)**	1 (reference)	**–**	1 (reference)	**–**
**Difference between the affected side and contralateral side of the MTE–MFC distance (>0.665 mm)**	19.125 (4.115–88.878)	<0.001*	15.384 (2.687–88.067)	0.002*
**Difference between the affected side and contralateral side of the MTFJ width (≥−0.575 mm)**	1 (reference)	**–**	1 (reference)	**–**
**Difference between the affected side and contralateral side of the MTFJ width (<−0.575 mm)**	14.450 (3.300–63.268)	<0.001*	50.846 (3.648–708.604)	0.003*

*Note*: ORs and 95% CI for MMPRT by X-ray measurements

*Abbreviations*: *MTE* medial tibial eminence, *MFC* medial femoral condyle, *MTFJ* medial tibial femoral joint, *MMPRT* medial meniscus posterior root tear, *OR* Odds ratio, *CI* confidence interval

^a^Adjusted for age, body mass index, and posterior tibial slope. *P < 0.05

### Reproducibility of X-ray and MRI measurements

Intra and interobserver measurements of the knee were consistent, as shown by the following ICCs: FTA: intraobserver, 0.867–0.922 and interobserver, 0.823–0.852; PTS: intraobserver, 0.811–0.952 and interobserver, 0.702–0.829; MTE–MFC distance: intraobserver, 0.818–0.983 and interobserver, 0.743–0.877; MTFJ width: intraobserver, 0.846–0.888 and interobserver, 0.821–0.880; and MRD: intraobserver, 0.951–0.981 and interobserver, 0.803–0.838.

## Discussion

In this pilot study of the potential of the non-weight-bearing tunnel view in X-ray diagnosis for the initial diagnosis of MMPRT, we found that the difference between the affected and contralateral sides in MTFJ width was significantly higher in the MMPRT group than in the non-MMPRT group. Further, the difference between sides in MTE–MFC distance was also significantly greater in the MMPRT group. On multivariate logistic regression analysis, differences between sides in MTFJ width of <−0.575 mm and in MTE–MFC distance of >0.665 mm were useful for predicting MMPRT. These results remained after adjustment for age, BMI, and PTS. Although preliminary, these findings suggest the potential of the non-weight-bearing tunnel view in X-ray imaging for the initial diagnosis of MMPRT.

To date, a number of useful X-ray imaging methods for osteoarthritis evaluation have been reported, including the Rosenberg view, the Camp–Coventry method, the Béclère method, and the Schuss view [2, 4]. Although these methods all demonstrate joint-space narrowing, the posterior aspect of the intercondylar notch, the inner posterior aspects of the medial and lateral femoral condyles, and the tibial spines and plateaus [1, 2, 14, 22], recent studies have not reached a consensus on which method is most useful for evaluating joint-space narrowing in osteoarthritis [16, 26]. Furthermore, few reports have described X-ray imaging methods that are useful for medial meniscus injury, especially for MMPRT. Recently, Kodama et al. [22] investigated the Rosenberg view and reported that MTFJ width was decreased and MTE–MFC distance increased when MMPRT occurred [15]. We obtained similar results in this study. Moreover, we found significant correlations between MMPRT and differences between the affected and contralateral sides in MTFJ width of <−0.575 mm and in MTE–MFC distance of >0.665 mm on multivariate logistic regression analysis. One study recommended a weight-bearing tunnel view for evaluation of osteoarthritis. Regardless, our present results are similar to those of a previous study using the Rosenberg view in MMPRT [15]. Although no study has yet compared weight-bearing and non-weight-bearing tunnel views for MMPRT, our present preliminary findings suggest that the non-weight-bearing tunnel view is useful in the early diagnosis of this condition, and warrants prospective evaluation.

It is known that when MMPRT occurs, the hooping action of the medial meniscus is disrupted, which in turn causes its extrusion. The stabilizer function of the meniscus is consequently disrupted and tibiofemoral contact pressure increases [1, 8]. In this study, there were significant differences in MRD between the MMPRT and non-MMPRT groups. In previous studies, flexion of the normal knee was shown to cause a rollback motion of the tibia with external rotation, during which the MFC moves medioposteriorly [19, 27], and medioposterior movement of the medial meniscus at 90° flexion [29]. Further, non-weight-bearing MRI in MMPRT patients showed that the medial meniscus moves further posteriorly (*P* < 0.0001) and medially (*P* = 0.05) at 90° flexion relative to 10° flexion [17]. We therefore speculate that knee flexion with MMPRT causes an MFC shift and joint-space narrowing due to posterior and medial dislocation of the medial meniscus. In fact, a cadaveric study on non-weight-bearing knees with 0°–90° flexion reported that 60° flexion had the highest medial translation of the MFC and medial compartment contact pressure as a result of loss of the buttress effect against the MFC [1]. These findings likely explain the decreased MTFJ width and increased MTE–MFC distance we observed on the affected side compared with the contralateral side in our MMPRT group.

Early detection of MMPRT is important in preventing the rapid progression of degenerative knee disease [5, 6]. The usefulness of the Rosenberg view in MMPRT has been reported [15]. However, the Rosenberg view requires patients with MMPRT who have knee pain to maintain a standing position with knee flexion of 45° for the duration of imaging. In contrast, the non-weight-bearing tunnel view evaluated in our present study allows the knee to be maintained in flexion without load, allowing X-rays to be taken without load-related pain. Allowing that confirmation in a larger study is required, these findings may suggest that this imaging method is more effective than the Rosenberg view in these patients.

This study has several limitations. First, the number of patients was inadequate for drawing any firm conclusions regarding the usefulness of the non-weight-bearing tunnel view for MMPRT patients. However, the Béclère method that we used is over an angle block and sandbag, and it is therefore a reproducible method. Moreover, all measurements have high intraobserver and interobserver reliability. Second, we could not analyze results for this view between patients with MMPRT and healthy controls. However, in this study, all patients are less degeneration and patient with non-MMPRT group had no meniscal tear. Finally, contralateral MMPRT could not be ruled out in all patients using images. However, no patient reported contralateral knee pain; thus, contralateral MMPRT was unlikely. Prospective analysis with other methods of X-ray imaging and a larger number of patients, including healthy controls, are warranted.

## Conclusions

This study shows that the non-weight-bearing tunnel view is a useful imaging method for initial diagnosis of MMPRT. Comparison of X-ray characteristics and MRI findings between the MMPRT and non-MMPRT groups showed significantly greater differences in MTFJ width and MTE–MFC distance between the affected and contralateral sides on non-weight-bearing tunnel view in the MMPRT group than in the non-MMPRT group. Prospective analysis is warranted.

## Data Availability

The datasets analyzed in the current study are available from the corresponding author on reasonable request.

## Abbreviations

BMI: Body mass index
FTA: Femorotibial angle
ICC: Intra-class correlation coefficients
MFC: Medial femoral condyle
MMPRT: Medial meniscus posterior root tear
MRD: Medial radial displacement
MRI: Magnetic resonance imaging
MTE: Medial tibial eminence
MTFJ: Medial tibiofemoral joint
PTS: Posterior tibial slope
